# Auditory observation of infant-directed speech by mothers: experience-dependent interaction between language and emotion in the basal ganglia

**DOI:** 10.3389/fnhum.2014.00907

**Published:** 2014-11-10

**Authors:** Yoshi-Taka Matsuda, Kenichi Ueno, Kang Cheng, Yukuo Konishi, Reiko Mazuka, Kazuo Okanoya

**Affiliations:** ^1^Okanoya Emotional Information Project, Exploratory Research for Advanced Technology (ERATO), Japan Science and Technology Agency (JST)Wako, Saitama, Japan; ^2^Cognition and Behavior Joint Research Laboratory, RIKEN Brain Science InstituteWako, Saitama, Japan; ^3^Laboratory for Language Development, RIKEN Brain Science InstituteWako, Saitama, Japan; ^4^Center for Baby Science, Doshisha UniversityKyoto, Japan; ^5^Support Unit for Functional MRI, RIKEN Brain Science InstituteWako-shi, Saitama, Japan; ^6^Department of Life Sciences, Graduate School of Arts and Sciences, The University of TokyoTokyo, Japan

**Keywords:** basal ganglia, emotion, experience dependency, functional magnetic resonance imaging (fMRI), infant-directed speech, language, maternal behavior

## Abstract

Adults address infants with a special speech register known as infant-directed speech (IDS), which conveys both linguistic and emotional information through its characteristic lexicon and exaggerated prosody (e.g., higher pitched, slower, and hyperarticulated). Although caregivers are known to regulate the usage of IDS (linguistic and emotional components) depending on their child’s development, the underlying neural substrates of this flexible modification are largely unknown. Here, using an auditory observation method and functional magnetic resonance imaging (fMRI) of four different groups of females, we revealed the experience-dependent influence of the emotional component on linguistic processing in the right caudate nucleus when mothers process IDS: (1) non-mothers, who do not use IDS regularly, showed no significant difference between IDS and adult-directed speech (ADS); (2) mothers with preverbal infants, who primarily use the emotional component of IDS, showed the main effect of the emotional component of IDS; (3) mothers with toddlers at the two-word stage, who use both linguistic and emotional components of IDS, showed an interaction between the linguistic and emotional components of IDS; and (4) mothers with school-age children, who use ADS rather than IDS toward their children, showed a tendency toward the main effect of ADS. The task that was most comparable to the naturalistic categories of IDS (i.e., explicit-language and implicit-emotion processing) recruited the right caudate nucleus, but it was not recruited in the control, less naturalistic condition (explicit-emotion and implicit-language processing). Our results indicate that the right caudate nucleus processes experience-and task-dependent interactions between language and emotion in mothers’ IDS.

## Introduction

Across languages and cultures, adults often use a speech register known as infant-directed speech (IDS) when talking to infants. IDS typically involves modifications of speech prosody, such as a higher pitch, an exaggerated intonation contour, and lexical, syntactic, and other modulations (Snow and Ferguson, 1977; Soderstrom, 2007; Saint-Georges et al., 2013). The lexical modifications often include a sizeable number of “baby-talk” words and frequently include onomatopoeia (Mazuka et al., 2008). These modifications draw infants’ attention to speech (Werker and McLeod, 1989; Cooper and Aslin, 1990; Fernald, 1991; Barker and Newman, 2004; Zangl and Mills, 2007) and may play a facilitating role in the emotional and linguistic development of infants (Thiessen et al., 2005; Vallabha et al., 2007; Kuhl and Rivera-Gaxiola, 2008; Taumoepeau and Ruffman, 2008). Recent neuroimaging studies support these IDS functions. The perception of IDS activated the orbitofrontal cortex and language areas in infants (Dehaene-Lambertz et al., 2002; Pena et al., 2003; Saito et al., 2007), where the affective and linguistic properties are encoded, respectively (Minagawa-Kawai et al., 2007, 2009). Interestingly, even when affective exaggeration is removed in IDS, formant-exaggeration alone could elicit enhanced neural activities in the infant brain (Zhang et al., 2011).

Because IDS conveys both affective and linguistic information towards infants (Kuhl et al., 1997; Trainor et al., 2000; Burnham et al., 2002), caregivers are known to regulate the proportion of the two types of information depending on the child’s level of development to obtain a sufficient reaction from developing infants (Snow and Ferguson, 1977; Saint-Georges et al., 2013). Emotionally biased IDS toward preverbal infants gradually shifts to both emotionally and linguistically balanced speech toward talking infants at approximately the two-word utterance stage (Penman et al., 1983; Kitamura and Burnham, 2003; Kajikawa et al., 2004; Amano et al., 2006; Ogura, 2006). As the infant’s language ability develops, IDS changes to a less emotional and linguistically more complex style. By the time caregivers are speaking to school-age children, the speech is comparable to adult-directed speech (ADS; Garnica, 1977). Little is known, however, as to what neural processes in adult caregivers encode this flexible modification of IDS. We previously reported the experience-dependent processing of IDS in language areas (Matsuda et al., 2011). Mothers with preverbal infants showed an enhanced activity of the emotional (prosodic) component of IDS. This group is known to use the emotional component of IDS more heavily compared with non-mothers or mothers with older children (Amano et al., 2006). Similarly, mothers with infants in the two-word utterance stage and mothers with preverbal infants showed an enhanced activity of the linguistic (lexical) component of IDS. These mother groups are known to use the linguistic component of IDS frequently (Ogura, 2006). Mothers with school-age children and non-mothers do not use emotional or linguistic components of IDS regularly and did not show enhanced activity. Thus, these results indicate the presence of use-dependent processing of IDS in the language areas, which reflects the current usage of IDS properties. However, this study only investigated the main effect of either the prosody (i.e., IDS prosody vs. ADS prosody) or lexicon (i.e., IDS lexicon vs. ADS lexicon) separately without thorough consideration of the mutual interaction between prosody and lexicon, i.e., (IDS prosody, ADS prosody) × (IDS lexicon, ADS lexicon). In the naturalistic environment, mothers use both prosodic and lexical properties simultaneously to communicate with their infants and regulate the proportion used depending on the child’s level of development (Snow and Ferguson, 1977; Saint-Georges et al., 2013); thus, it remains unclear as to what neural process encodes the dynamic interaction and integration between the two types of IDS depending on the parenting experience.

**Figure 1:**
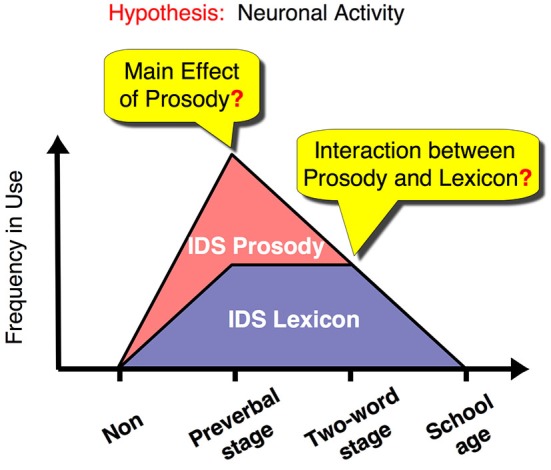
**Dynamic changes in IDS components in relation to child development and our proposed hypothesis for the changes in underlying neural activity**. The graph illustrates the frequency of use of caregivers’ IDS for prosody and lexicon (red and purple area graphs, respectively). We suggest a neural representation that reflects this dynamic change in IDS and therefore fulfills all of the following conditions: (1) Mothers with preverbal infants use IDS prosody, rather than IDS lexicon, as a main channel for their communication and show a main effect for processing IDS prosody. (2) Mothers with toddlers in the two-word utterance stage use both IDS prosody and lexicon equally and show an interaction effect between the two components. (3) Non-mothers and mothers with children in the first year of elementary school do not use IDS routinely and do not show a main effect or an interaction effect between lexical and prosodic components. Non: non-mothers, Preverbal stage: mothers with preverbal infants, Two-word stage: mothers with toddlers in the two-word utterance stage, School age: mothers with children in the first year of elementary school.

Here, we used functional magnetic resonance imaging (fMRI) to investigate the specific neural substrates that showed a dynamic change corresponding to the interaction between emotion (prosody) and language (lexicon) depending on the child’s developmental stage (see time course in Figure 1). To address this issue, we reanalyzed our previous data (Matsuda et al., 2011), which comprised four groups of female adult participants, including women without children and women with children in different stages of language development: (1) non-mothers; (2) mothers with preverbal infants; (3) mothers with toddlers speaking in two-word utterances; and (4) mothers with children in elementary school. Each group of mothers represents a distinct stage in the time course of IDS usage. Of the four groups, the mothers with preverbal infants are the heaviest users of prosodic IDS (Amano et al., 2006). Around the time children are in the two-word utterance stage, mothers proportionally suppress prosodic modulation (Amano et al., 2006), while they continue to use lexical IDS (Ogura, 2006). This is the period when emotionally and linguistically balanced IDS is produced. Subsequently, mothers virtually cease to use both prosodic and lexical IDS toward elementary-school children. In this study, non-mothers were used as a control group.

The experiment consisted of two separate tasks, which contrasted a more naturalistic environment with a less naturalistic control condition in regard to the actual usage of IDS. As caregivers are known to address infants with a conscious conversational and sub-conscious emotional speech style in IDS (Snow and Ferguson, 1977), the explicit-linguistic and implicit-emotional processing (the lexicon task) resembled a naturalistic condition. However, the explicit-emotional and implicit-linguistic processing (the prosody task) was different from the actual environment and served as a control condition in this study.

**Figure 2:**
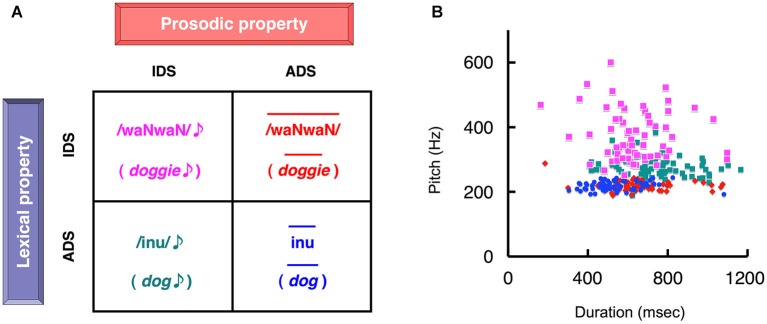
**The four types of auditory stimuli presented in the two separate experimental tasks. (A)** Subjects reported whether the stimulus was infant-directed speech (IDS) or adult-directed speech (ADS), attending selectively to either the lexicon (lexicon task) or prosody (prosody task). This decision was followed by the mental rehearsal of the percept during the inter-stimulus interval. The two-by-two factorial design created four word sets of stimuli that contained semantically equivalent words. Each element of the set differed in the combination of lexicon (IDS or ADS) and prosody (IDS or ADS). The figure represents examples of four different speech sounds that are the semantic equivalent of “dog”. In the lexicon task, the stimuli in the upper and lower rows correspond to IDS and ADS, respectively, whereas in the prosody task the left and right columns correspond to IDS and ADS stimuli, respectively. **(B)** A scatter plot of duration vs. pitch (fundamental frequency) for the 64 sets of stimuli used (Matsuda et al., 2011). The duration and pitch of each stimulus were extracted as acoustic parameters. Colors correspond to the types of stimuli assigned in **(A)**. Note that each distinct cluster reflected the phonetically inherent characteristics of IDS and ADS (reproduced from Matsuda et al., 2011).

In the lexicon task (naturalistic condition), the subjects judged whether auditory stimuli were IDS or ADS by focusing on the word while ignoring prosody (Figure 2A). In the prosody task (control condition), the subjects judged whether auditory stimuli were IDS or ADS by focusing on only prosody while ignoring word content (Figure 2A).

We used an IDS perception experiment rather than a production experiment to avoid the effect of head movement on fMRI signal. One property of IDS production is hyperarticulation, and speakers move their mouths more dynamically as opposed to ADS production. This hyperarticulation could inevitably cause head movement and subsequently decrease the signal-to-noise ratio of the fMRI signal. Owing to a fine-tuned sensorimotor “resonance” system, which develops as a consequence of extensive motor practice (Aglioti et al., 2008), we conducted an IDS perception experiment rather than a production experiment.

## Methods

All data sets were obtained as part of our previous study (Matsuda et al., 2011) and were reanalyzed with different methods to address the issues raised in the current study.

### Participants

Sixty-nine participants comprised four different groups, which included 15 non-mothers, 20 first-time mothers with preverbal infants, 16 first-time mothers with toddlers who spoke two-word utterances, and 18 mothers with children in the first grade. We confirmed the presence of two-word utterances in each toddler via parental report on the toddler form of the MacArthur Communicative Development Inventory (Watamaki and Ogura, 2004). No parent was single, and all mothers were the primary caregivers. All subjects were neurologically normal, right-handed Japanese speakers, and no one was professionally involved in infant care. All groups were matched for education and verbal IQ. All subjects provided informed consent according to the procedures approved by the RIKEN Brain Science Institute Ethics Committee and the Functional MRI Safety and Ethics Committee in Wako (for further details, see Matsuda et al., 2011).

### Auditory stimuli

The stimulus sets were the same as those used in our previous study (Matsuda et al., 2011). Briefly, we prepared 64 pairs of word items with semantically equivalent meanings but different vocabularies: words utilized in the context of (i) IDS and (ii) ADS, e.g., /waNwaN/ (doggie) and /inu/ (dog), respectively. The stimuli were recordings of a professional actress speaking each word in two prosodic conditions: (i) IDS prosody in the presence of her preverbal infant son and (ii) ADS prosody directed at an adult, which resulted in 256 stimuli (64 lexical items × 2 linguistic conditions × 2 prosodic conditions). This balanced two-by-two factorial design, which used lexicon or prosody to identify an item as IDS or ADS, resulted in four different types of stimuli (Figure 2A). Congruous ADS speech (ADS words with ADS prosody) had a shorter duration and lower pitch, congruous IDS speech (IDS words with IDS prosody) had a varying duration and higher pitch, and the two incongruous speech-stimuli (IDS words with ADS prosody and ADS words with IDS prosody) fell between the two other forms of speech (Figure 2B). Additional details have been provided in our previous work (Matsuda et al., 2011).

### Experimental design

Each subject performed two separate tasks (prosody and lexicon). In the prosody task, the subjects judged whether binaurally presented auditory stimuli were IDS or ADS by focusing on only prosody while ignoring word content. In the lexicon task, the subjects judged whether the stimuli were IDS or ADS by focusing on the word while ignoring prosody. The participants were asked to respond as quickly and as accurately as possible after the recognition of each stimulus by pressing a button with either the left or right hand. Within each task, 128 words were presented in quasi-random order. The order of the two tasks was counterbalanced across subjects (for additional details, see Matsuda et al., 2011).

### MRI scanning procedure

fMRI experiments were performed on a 4T Agilent whole-body MRI system (Agilent Inc., Santa Clara, CA, USA). Twenty-three axial slices (24-cm FOV, 64 × 64 matrix, 5-mm thickness, 0-mm gap) parallel to the anterior commissure-posterior commissure (AC-PC) plane were acquired using a two-shot Echo Planar Imaging (EPI) pulse sequence (volume TR 2,600 ms, TE 25 ms, flip angle 40°) for the two functional runs; each run consisted of 298 volumes. Prior to and between the functional runs, a set of high-resolution (1 mm^3^) and low-resolution (1.72 mm^3^) whole-brain anatomical images were acquired using a magnetization-prepared 3D FLASH (fast low-angle shot) pulse sequence (for additional details, see Matsuda et al., 2011).

### fMRI data analysis

EPI image reconstruction was followed by respiratory-and cardiac-fluctuation removal using a previously described retrospective estimation and correction method (Cheng et al., 2001). The data were then preprocessed and analyzed using the BrainVoyager QX software package (Brain Innovation, Maastricht, Netherlands).

After the transformation of each subject’s registered functional images into the Talairach space (Talairach and Tournoux, 1988), whole-brain activation maps were obtained using a standard voxel-wise general linear model (GLM) at the single-subject level. To extract the brain regions involved in the perceptual processing of each speech sound (IDS or ADS), multiple regressors were generated by convolving a boxcar representation of each type of stimulus-presentation period prior to the subject response with a theoretical two-gamma hemodynamic response function with an event-related design. A second-stage random-effect analysis (RFX) was then performed across subjects. Activated clusters with less than 281 mm^3^ (4 voxels; 1 voxel = 3.75 × 3.75 × 5 mm^3^) were omitted from our results (for additional details, see Matsuda et al., 2011).

To compare contrasted activations among the subject groups, an analysis of variance (ANOVA) was conducted with subject-wise contrast, i.e., a 2 (prosody type) × 2 (lexicon type) design, as the repeated-measures factor and group as the between-subject factor for the prosody or lexicon tasks. We had a strong a priori hypothesis regarding how different groups will show different activation patterns that would show a dynamic change corresponding to the interaction between emotion (prosody) and language (lexicon) depending on the child’s developmental stage (Figure 1). Thus, we conducted a three-way ANOVA (2 (prosody type) × 2 (lexicon type) × 4 (group type)) for each task to determine the brain areas with a significant “interaction” (*q* < 0.05, False Discovery Rate or FDR correction for whole brain (Genovese et al., 2002)). Then, we confirmed whether each detected brain area (region of interest or ROI) satisfied our hypothesized patterns of activation (Figure 1) using *post hoc* comparisons between the subject groups. Because we did not assume bias in the selection of each ROI, the *post hoc* comparisons are valid and different from a circular analysis (Kriegeskorte et al., 2009).

## Results

### Behavioral results

We analyzed the reaction time and accuracy of judgment for IDS or ADS stimuli in the lexicon and prosody tasks for each participant. The reaction time was measured from the end point of each stimulus presentation to compensate for stimulus time variations. The percent correct data were arcsine transformed to stabilize variance when we conducted statistical analysis.

First, the difference of task difficulty was investigated by direct comparison between the lexicon and prosody tasks across subject groups and stimulus types. Analysis of the reaction time data revealed a significant difference between the tasks (one-way ANOVA, 2 tasks) (*F*_(1,68)_ = 20.02, *p* < 0.001), which indicates faster responses to the prosodic judgment (mean, 622 ms) compared with the lexical judgment (mean, 712 ms) across subject groups. The prosody information, e.g., voice pitch, was included from the initial part of the stimuli, and thus subjects could detect and discriminate the prosody component of the stimuli more quickly. However, the lexical information enabled subjects to understand meanings when they listen to each stimulus to the end, and thus the subject’s reaction time might be slower than in the prosody-judgment task. There was no significant difference among the subject groups (*F*_(3,65)_ = 0.81, *p* = 0.49). Analysis of the accuracy revealed a subtle but significant difference between the tasks (one-way ANOVA, 2 tasks) (*F*_(1,68)_ = 5.01, *p* < 0.05), which indicates that although the participants responded more accurately in the lexicon task (mean, 98.2%) compared with the prosody task (mean, 97.5%), the stimuli in both tasks were easily recognized as IDS or ADS. There was no significant difference among the subject groups (*F*_(3,65)_ = 2.16, *p* = 0.10).

Next, we investigated subject-group differences in the reaction time and accuracy by considering the stimulus types. We conducted ANOVAs that treated stimulus types of *Lexicon* (IDS, ADS) and *Prosody* (IDS, ADS) as the repeated measure factors and *Experience* as the between-subject factor (i.e., 2 lexicon-types × 2 prosody-types × 4 groups) in the lexicon-judgment and prosody-judgment tasks (the lexicon-judgment task is shown in Table 1, and the prosody-judgment task shown in Table 2).

**Table 1:** **Performance in the lexicon task for each subject group**.

	Mean reaction time (SD) (msec)		Mean accuracy (SD) (%)	
	Lexical ADS	Lexical IDS	Lexical ADS	Lexical IDS
**Non-mothers (*n* = 15)**
Prosodic ADS	652.8 (146.2)	652.8 (146.1)	97.9 (3.7)	93.5 (5.8)
Prosodic IDS	604.7 (200.0)	615.4 (147.7)	97.9 (3.3)	97.9 (3.7)
**Mothers with preverbal infants (*n* = 20)**
	758.3 (195.5)	747.8 (203.2)	98.9 (1.8)	96.6 (4.2)
	704.2 (248.9)	724.0 (243.2)	98.5 (2.9)	98.6 (2.1)
**Mothers with toddlers in the two-word utterance stage (*n* = 16)**
	788.3 (174.6)	787.8 (231.3)	99.2 (1.4)	98.2 (2.0)
	743.0 (200.9)	737.3 (217.9)	98.2 (3.2)	99.2 (1.8)
**Mothers with children in the first year of elementary school (*n* = 18)**
	727.4 (130.7)	729.4 (137.2)	100 (0)	99.0 (2.1)
	682.8 (142.3)	689.4 (144.3)	99.3 (1.7)	98.3 (3.1)

*Note: Reaction time was measured from the end point of each stimulus presentation to compensate for stimulus time variations. ADS: adult-directed speech, IDS: infant-directed speech, SD: standard deviation*.

**Table 2:** **Performance in the prosody task for each subject group**.

	Mean reaction time (SD) (msec)		Mean accuracy (SD) (%)	
	Prosodic ADS	Prosodic IDS	Prosodic ADS	Prosodic IDS
**Non-mothers (*n* = 15)**
Lexical ADS	609.2 (146.2)	557.9 (150.8)	99.2 (1.9)	98.1 (2.0)
Lexical IDS	584.0 (185.7)	520.1 (141.3)	94.8 (3.0)	96.9 (4.3)
**Mothers with preverbal infants (*n* = 20)**
	677.0 (225.9)	652.6 (224.5)	99.7 (1.0)	98.0 (3.1)
	659.7 (244.0)	650.8 (254.2)	96.1 (4.2)	97.8 (3.8)
**Mothers with toddlers in the two-word utterance stage (*n* = 16)**
	670.8 (208.1)	634.2 (235.5)	99.0 (3.2)	98.8 (1.9)
	684.2 (226.1)	583.7 (227.6)	94.7 (5.3)	97.4 (3.6)
**Mothers with children in the first year of elementary school (*n* = 18)**
	607.3 (143.6)	628.0 (168.5)	99.0 (2.1)	98.6 (2.4)
	597.2 (141.0)	590.7 (148.0)	95.0 (4.4)	97.0 (5.0)

*Note: Reaction time was measured from the end point of each stimulus presentation to compensate for stimulus time variations. ADS: adult-directed speech, IDS: infant-directed speech, SD: standard deviation*.

In the lexicon-judgment task, the reaction time did not show a significant main effect of subject group (*F*_(3,65)_ = 1.73, *p* = 0.17), but the accuracy did show a significant main effect (*F*_(3,65)_ = 3.59, *p* < 0.05). Slightly lower accuracy on the lexicon-judgment task in the non-mothers (mean, 96.8%) compared with the three groups of mothers (mean, 98.6%) may reflect the relatively little experience non-mothers have with IDS. There were no significant interactions between stimulus types and groups (i.e., 2 lexicon-types × 4 groups; 2 prosody-types × 4 groups; 2 lexicon-types × 2 prosody-types × 4 groups) for either reaction time or accuracy (*F*_(3,65)_ ≤ 2.70, *p* > 0.05 for all cases).

In the prosody-judgment task, neither reaction time (*F*_(3,65)_ = 0.79, *p* = 0.50) nor accuracy (*F*_(3,65)_ = 0.58, *p* = 0.63) showed a significant main effect of subject group. There were no significant interactions between stimulus types and groups (i.e., 2 lexicon-types × 4 groups; 2 prosody-types × 4 groups; 2 lexicon-types × 2 prosody-types × 4 groups) for either reaction time or accuracy (*F*_(3,65)_ ≤ 1.49, *p* > 0.22 for all cases) with the exception of an interaction between prosody-types and subject groups in reaction time (2 prosody-types × 4 groups, (*F*_(3,65)_ = 3.21, *p* < 0.05)). Slightly different reaction times between IDS-vs. ADS-prosody judgment were observed in the prosody task in the mothers with school-age children (609 ms to IDS prosody; 602 ms to ADS prosody) compared with the other three groups (600 ms to IDS prosody; 647 ms to ADS prosody), which showed the mothers’ heightened sensitivity to ADS prosody. These differences may reflect the age of the mothers, who were the eldest group, rather than the experience with their children.

Behavioral results of the reaction time were essentially unchanged after removal of outliers beyond 2 × standard deviation (SD) of the individual participant’s reaction-time distributions in each group (2 participants; one of mothers of preverbal infants and one of mothers with school-age children) (Ratcliff, 1993). There were no outliers in the behavioral data of the accuracy.

Overall, our behavioral results indicate that attentional levels were approximately the same among the four subject groups.

### Functional imaging results

To determine the brain sites that are specifically involved in the IDS vs. ADS evaluation of word meaning and prosody, we contrasted the lexicon and prosody tasks directly. This contrast failed to reveal significant differences, which suggests that both tasks recruit topographically similar brain structures (data not shown).

**Figure 3:**
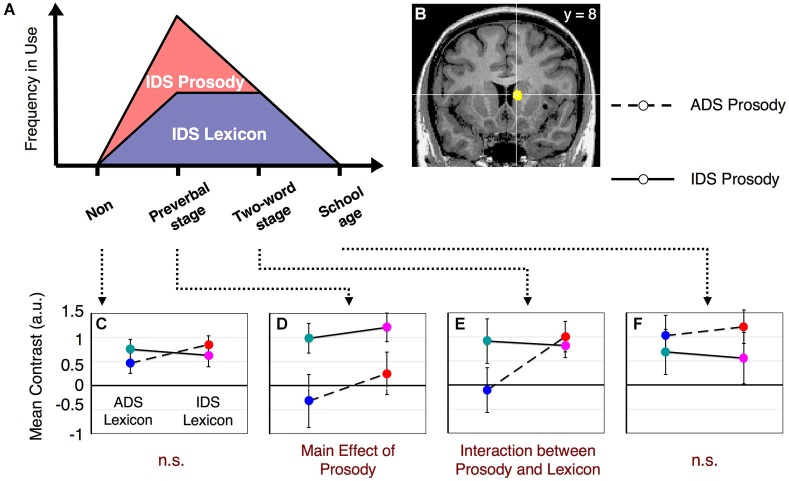
**fMRI results from the lexicon task**. The right caudate nucleus fulfills our hypothesized patterns of activation suggested in Figure 1. **(A)** The frequency of use of caregivers’ IDS for prosody and lexicon (the same graph as shown in Figure 1). **(B)** The center of the right caudate nucleus [Talairach coordinates (*x*, *y*, *z*) = (9, 8, 10)]. **(C)** Results from non-mothers. No significant main effect or interaction effect was observed. **(D)** Results from mothers with preverbal infants. There was a main effect of IDS prosody (*t*_(19)_ = 2.29, *p* < 0.05). **(E)** Results from mothers with toddlers in the two-word utterance stage. There was an interaction effect (*F*_(1,15)_= 76.19, *p* < 0.001). **(F)** Results from mothers with children in the first year of elementary school. No significant main effect or interaction effect was observed. The error bars indicate standard errors. Colors correspond to the types of stimuli assigned in Figure 2A.

**Figure 4:**
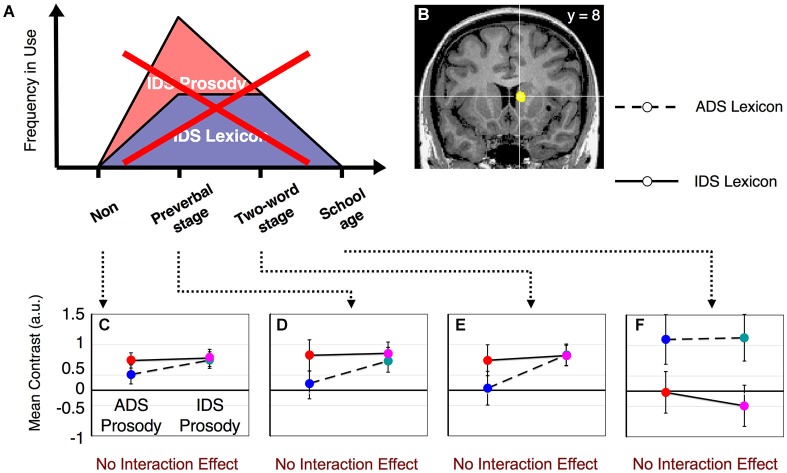
**fMRI results from the prosody task**. The right caudate nucleus does NOT fulfill our hypothesized patterns of activation when the prosody task is performed. No significant differences were observed among the subject groups in the interaction effect between lexicon and prosody (*F*_(3,65)_ = 0.58, *p* > 0.67). **(A)** The same graph as shown in Figure 1. **(B)** The same area of the right caudate nucleus as shown in Figure 3B. The results shown in **(C–F)** were from non-mothers **(C)**, mothers with preverbal infants **(D)**, mothers with toddlers in the two-word utterance stage **(E)** and mothers with children in the first year of elementary school **(F)**. The error bars indicate standard errors. Colors correspond to the types of stimuli assigned in Figure 2A.

Main effects and interactions of both the lexicon and prosody tasks in each subject group have been previously reported (Matsuda et al., 2011). In this study, we sought to identify the neural substrates that showed a dynamic change corresponding to the interaction between prosody-type and lexicon-type stimuli depending on the child’s developmental stage (Figure 1). By conducting a 2 (lexicon type) × 2 (prosody type) × 4 (group type) three-way ANOVA in the lexicon-judgment and prosody-judgment tasks, we identified three brain areas with significant interactions (*q* < 0.05, FDR) in the lexicon-judgment task, but none in the prosody-judgment task. These areas included the right caudate nucleus [Talairach coordinates (*x*, *y*, *z*): 9, 8, 10], the medial prefrontal cortex (mPFC) [5, 37, 9], and the right dorsolateral prefrontal cortex [35, 46, 17]. Within the three different areas, only the right caudate nucleus satisfied our hypothesized patterns of the experience-dependent behavior (Figure 3B). *Post hoc* comparisons among subject groups showed that the right caudate nucleus met the following four conditions in the lexicon-judgment task, i.e., a 2 (lexicon type) × 2 (prosody type) ANOVA for each subject group (Figures 3C–F): (1) non-mothers showed neither significant main effects (lexicon-type (IDS vs. ADS) and prosody-type (IDS vs. ADS)) nor interaction (2 (lexicon type) × 2 (prosody type)) (*F*_(1,14)_ < 3.19, *p* > 0.05 for all cases); (2) mothers with preverbal infants showed main effects of prosody type (*F*_(1,19)_ = 4.39, *p* < 0.05; mean of IDS prosody = 1.10, mean of ADS prosody = −0.03), and there were no significant main effects of lexicon type or interaction (2 (lexicon type) × 2 (prosody type)); (3) mothers with toddlers at the two-word stage showed interaction effects between lexicon and prosody types, where enhanced activities were evoked by either the lexical or prosodic IDS or by the combination of the two (*F*_(1,15)_ = 76.19, *p* < 0.001; mean of IDS lexicon and IDS prosody = 0.82, mean of IDS lexicon and ADS prosody = 1.01, mean of ADS lexicon and IDS prosody = 0.91, mean of ADS lexicon and ADS prosody = −0.11); and (4) mothers with school-age children showed no significant main effects (lexicon-type (IDS vs. ADS) and prosody-type (IDS vs. ADS)) nor interaction (2 (lexicon type) × 2 (prosody type)) (*F*_(1,17)_ < 0.77, *p* > 0.39 for all cases). This experience-dependent behavior in the caudate nucleus was observed in the lexicon task (explicit-lexicon and implicit-prosody processing), but not in the prosody task (explicit-prosody and implicit-lexicon processing). Among the subject groups, the right caudate nucleus did not show significant differences in the interaction effect between lexicon and prosody types in the prosody task (a 2 (lexicon type) × 2 (prosody type) × 4 (group type) ANOVA, *F*_(3,65)_ = 0.58, *p* = 0.67) (Figure 4). The mothers with school-age children showed a tendency toward different activity of lexicon type (IDS lexicon vs. ADS lexicon) compared with the other subject groups, but this finding was not significant among the subject groups (a 2 (lexicon type) × 4 (group type) ANOVA, *F*_(3,65)_ = 0.58, *p* = 0.67).

Although the medial and dorsolateral prefrontal cortices showed a significant interaction between lexicon, prosody and subject groups (a 2 (lexicon type) × 2 (prosody type) × 4 (group type) ANOVA) in the lexicon task, these areas did not meet our hypothesized patterns of activation (Figure 1). The main effect and the interaction were not significant in each subject group (*F*_(3,65)_ < 2.09, *p* > 0.1 for all cases).

To summarize, these results indicate that the ongoing use of IDS is reflected in the explicit lexicon and implicit prosody processing of IDS in the right caudate nucleus.

## Discussion

We determined that the right caudate nucleus is a neural substrate for the experience-dependent changes in the interaction between language and emotion in IDS processing. The task, which is close to the naturalistic environment of IDS usage, activated the right caudate nucleus in an experience-dependent manner as opposed to the control, less naturalistic condition, i.e., the right caudate nucleus is sensitive to the ongoing use of IDS components (prosody, lexicon or both), and provide neurophysiological evidence supporting IDS as a flexible, goal-directed behavior in social communication.

It is of interest to note that even though all participants performed the tasks in similar fashions (Tables 1, 2), the blood oxygen level dependent (BOLD) signals differed dramatically among the subject groups. This observation suggests that signal differences were not the result of difficulties in performing the tasks (e.g., delayed response or low accuracy) (Binder et al., 2004), but rather they were caused by the increase in sensitivity in the caudate nucleus.

While simple auditory perception and subsequent IDS or ADS judgment were required as experimental tasks, we could indirectly evoke the participants’ motor-related response to IDS by auditory observation of the IDS stimuli, which were uttered by another caregiver. We previously showed that a mother’s speech-related motor circuit is recruited by the perception of IDS in an experience-dependent manner (Matsuda et al., 2011). The observation of specific actions performed by other individuals appears to be linked to a covert simulation of well-trained motor activation. This fine-tuned sensorimotor “resonance” system develops as a consequence of extensive motor practice (Aglioti et al., 2008). Recent studies using transcranial magnetic stimulation demonstrated that stimulation of the production-related motor cortex resulted in an altered sensory-perception performance (Gough et al., 2005; Meister et al., 2007; D’Ausilio et al., 2009). This finding confirms the idea that the neural circuitry in motor areas is functionally and directly connected to perception processing. Auditory perception of IDS was expected to implicate the mothers’ motor system for IDS. Therefore we employed this use-dependent resonance effect as a functional probe of the motor response to IDS.

### IDS processing as a goal-directed behavior

IDS is the speech-related motor response to an infant, and its goal is to encourage and communicate with infants. Caregivers flexibly regulate their own IDS to obtain a sufficient reaction (outcome) from developing infants, and hence, the contents of IDS dynamically change through the modification of the ratio between the linguistic and emotional components. This flexibility makes IDS distinct from a habit system of stimulus–response learning or Pavlovian conditioning, which involves passive learning of stimulus–outcome associations. In the scheme of a goal-directed action with stimulus–response–outcome association learning in the mother-infant interplay, we revealed experience-dependent changes in IDS processing in the interaction between prosody and lexicon in the right caudate nucleus. The neural representations of the ongoing use and regulation of IDS are consistent with the known functions in this area, i.e., flexible goal-directed actions (Yin et al., 2005a,b; Yin and Knowlton, 2006). Beyond the arbitrary association learning of stimulus–response–outcome conditions identified in human (Valentin et al., 2007; Xue et al., 2008; de Wit et al., 2009) and animal studies (Miyachi et al., 1997, 2002; Yin et al., 2005a,b, 2009), our results demonstrate that the caudate nucleus is involved in naturalistic goal-directed behavior in social communication, such as the mother-infant interaction.

Previous studies have shown that during motor and procedural learning, changes in neural activity occur in the striatum, which is the major input center of the basal ganglia (Jenkins et al., 1994; Doyon et al., 1996; Carelli et al., 1997; Ungerleider et al., 2002; Brasted and Wise, 2004; Barnes et al., 2005). Several studies have also shown that different striatal circuits and processes are engaged during the early and late phases of skill learning (Miyachi et al., 1997, 2002; Costa et al., 2004; Yin et al., 2009). The dorsomedial or associative striatum (the caudate nucleus in primates), which receives input primarily from the association cortices, such as the PFC (McGeorge and Faull, 1989; Voorn et al., 2004), appears to be preferentially involved in the initial stages of visuomotor learning and during the rapid acquisition of action-outcome contingencies (Miyachi et al., 1997, 2002; Yin et al., 2005b). However, the dorsolateral or sensorimotor striatum (the putamen in primates), which receives inputs from the sensorimotor cortex (McGeorge and Faull, 1989; Voorn et al., 2004), is critical for the more gradual acquisition of habitual and automatic behaviors (Miyachi et al., 1997, 2002; Yin et al., 2004). Our results observed in the caudate nucleus are consistent with its function in the early phases of skill learning, i.e., flexible action-outcome contingencies in goal-directed behavior.

Our auditory observation paradigm of IDS may enable caregivers to recall the outcome (infant’s reaction) indirectly rather than having a direct and immediate outcome. The anticipation of peak emotion (or “chills”) activates the right caudate nucleus when human subjects listen to their own favorite music, while the experience of the peak activates the nucleus accumbens (Salimpoor et al., 2011). In our study, the experience-dependent activation in the right caudate nucleus was observed only in the lexicon task that required the explicit processing of lexicon and implicit processing of prosody of IDS. This lexicon task is more comparable to the naturalistic environment of IDS perception compared with the prosody task (explicit-prosody and implicit-lexicon processing of IDS). As caregivers are known to address infants with a conscious conversational and sub-conscious emotional speech style in IDS (Snow and Ferguson, 1977), the resemblance of the lexicon task to naturalistic IDS may easily induce the anticipation of the outcome, i.e., the infant’s reaction. The results further suggest that the right caudate nucleus represents experience-dependent changes in the outcome anticipation for caregivers, which co-varies with the speech style of IDS along with child development.

However, there might be alternative possibilities other than goal-directed behavior and/or prolonged sensorimotor tuning, but simply a different strategy for performing the tasks. One alternative possibility is that mothers with IDS experience automatically anticipated the infant’s reaction. The right caudate activation might be attributable to some subject groups imagining infants’ reactions and others not doing so. However, our fMRI results of group comparisons did not show our hypothesized pattern of activity (Figure 1) in either the visual areas or memory-related areas (e.g., hippocampus), which indicate that there were no differences among subject groups in imaging and/or remembering of infants’ reaction by the auditory stimuli. One may further claim that even non-mothers would also activate the right caudate nucleus if they were asked to imagine an infant’s reaction. We exclude the possibility of the readiness of imaging an infant’s reaction by auditory stimuli through parental experience. This conclusion follows from our finding that non-mothers and mothers with school-age children showed similar responses in the right caudate nucleus (Figure 3). Another possibility is a group difference in threshold to suppress the prosody information, which was spoken by another mother. Mothers with preverbal infants or toddlers frequently use IDS prosody in their own way including individual differences, and thus the auditory observation of other’s IDS prosody might interfere and by necessity suppress the processing of their own IDS to properly conduct the lexicon-judgment task with emotionally charged stimuli. If the suppression threshold was lower, the reaction time should be slower in these subject groups, but this was not the case. As we mentioned in the results section for the behavioral data, the between-subject comparison did not show a significant interaction with stimulus-type differences in the lexicon-judgment task.

### Asymmetrical interaction between language and emotion in IDS

Notably, the right caudate nucleus showed distinct activity that depended on the tasks that the subjects performed. The caudate nucleus exhibited experience-*dependent* processing in the lexicon task, while it displayed experience-*independent* processing in the prosody task. These differences between the two tasks suggest an asymmetrical interaction between lexical and prosodic processing in IDS, i.e., prosodic interference to lexical processing differs from lexical interference to prosodic processing.

Our results may lead us to reconsider the three generally accepted functions of IDS (Grieser and Kuhl, 1988; Cooper et al., 1997; Singh et al., 2002). First, IDS may attract and maintain infants’ attention. Second, it may communicate positive emotion between a caregiver and an infant. Third, it may facilitate language acquisition. These three functions may coexist during infant development, with the attentional and emotional functions dominating during early infancy, and the linguistic function gaining importance as the infant progresses in the language learning process (Song et al., 2010). Our study suggests that this dynamic shift in IDS functions results from explicit processing of language communication rather than the intended emotional communication. As revealed in this study, only explicit language processing (i.e., the lexicon task) enabled this shift in the course of the infant’s development. We contend that the emotional function during early infancy is an implicit by-product induced by the intention to produce language communication with preverbal infants, which is, in effect, an explicit goal-directed action of caregivers.

## Conflict of interest statement

The authors declare that the research was conducted in the absence of any commercial or financial relationships that could be construed as a potential conflict of interest.
